# Promoting the propensity for blood donation through the understanding of its determinants

**DOI:** 10.1186/s12913-021-06134-8

**Published:** 2021-02-08

**Authors:** Roberta Guglielmetti Mugion, Maria Giovina Pasca, Laura Di Di Pietro, Maria Francesca Renzi

**Affiliations:** University of Roma Tre, Via Silvio D’Amico 77, 00145 Rome, Italy

**Keywords:** Blood donation, Theory of planned behaviour, Donors, Non-donors, Antecedents, SEM, WOM

## Abstract

**Background:**

The paper aims to understand the main antecedents related to the blood donation propensity related to both donors and non-donors. With our research, we will analyse the two perspectives to identify similarities and differences concentrating on the Italian context. Blood is a vital resource that strongly affects every national healthcare system’s efficacy and sustainability and the system’s ability to achieve the goal of universal coverage.

**Methods:**

The purpose of this paper is to understand the main antecedents of citizens’ blood donation intention and the propensity to encourage communication about blood donation among both donors and non-donors. The Theory of Planned Behaviour is adopted as a theoretical lens. An empirical investigation was performed in Italy, adopting a mixed methods research design. First, a qualitative analysis was carried out through 30 in-depth interviews. Then, a survey was used to quantitatively investigate the intention to donate among both donors (*N* = 173) and non-donors (*N* = 87). A conceptual model was developed and tested through Structural Equation Modelling, developing a multi-group approach.

**Results:**

The present study confirms the relations proposed by the Theory of Planned Behaviour, even though some differences between the two groups are shown. The construct Information and Communication is crucial for donors, non-donors, whereas for non-donor inhibitors is vital. Service quality has an impact on the propensity to recommend and communicate the value of blood donation.

**Conclusion:**

This paper reveals the main differences between donor and non-donor perspectives. Fruitful insights for enhancing blood donation awareness are provided.

**Supplementary Information:**

The online version contains supplementary material available at 10.1186/s12913-021-06134-8.

## Background

Blood is a vital healthcare resource that strongly affects every national healthcare system’s efficacy and sustainability and the system’s ability to achieve the goal of universal health coverage [1]. Unfortunately, blood is a limited resource that cannot be reproduced and presents a little lifecycle from donation to utilisation [2]. National governments must raise awareness of the phenomenon of blood donation by ensuring access to sufficient and safe blood. In Italy, donating blood is a voluntary, unpaid activity and anonymous as it is not possible to “address” the donated blood for ethical and safety reasons. Hence, it may be defined as a social activity that individuals carry out to contribute to human well-being [3] positively.

Although the WHO highlights that from 2008 to 2015, an increase of 11.6 million blood donations from voluntary non-remunerated donors was detected [4], blood demand is continuously increasing. It will continue to grow in the next decades due to both stricter parameters to assure the safety of collected blood [5] and the broader blood demand coming from, the older population [6]. As pointed out by Greinacher et al., all these aspects could generate a dangerous shortage of available blood [7]. Therefore, it is crucial to incentivise an increase in the number of citizens who voluntarily decide to contribute to donation, thus overcoming the deficiency of available blood and contributing to community well-being. In order to build a stable base of blood donors, there are the following two main strategies: i) recruiting new donors, particularly among young generations, and ii) retaining donors and increasing their frequency of donation [8, 9].

As stated by the WHO and the International Federation of Red Cross and Red Crescent Societies (IFRC), “*building a sustainable base of safe blood donors requires a long-term approach, that requires not only the establishment of an effective voluntary blood donor program but also an improved public awareness of the importance of blood donation as a social norm*” [10].

Abbasi et al. [11] pointed out that “to meet the requirements for blood in developing countries, 1% of the population needs to donate blood”. They identified a substantial inequity in the attitude towards voluntary blood donation between developed and developing countries.

It is essential to emphasise that blood availability is fundamental in first aid services, surgery, the treatment of certain diseases (e.g., oncological diseases), transplants and transfusions. Thus, self-sufficiency is undoubtedly a crucial element both at the regional and individual hospitals, and hospitals have an increasing need for blood donations [3, 12, 13].

In the Italian healthcare sector, blood donation is a complex process in which several public and private stakeholders are involved, including public hospitals, donation associations, private foundations and citizens. Italian government states that blood donation is an unpaid activity for the engaged donors, which can only be compensated with low-cost services, small tokens, refreshments such as free breakfast or a discount voucher for theatres and cinemas. For this reason, legal action may be taken against anyone who donates blood for money (Art. 22, Law 219/2005). Hence the donation is considered a free, conscious and non-profit activity, carried out by voluntary non-remunerated blood donors (VNRD). The Italian Blood Volunteers Association (AVIS) have defined ethical requirements for becoming and being donors. To become a donor, it is necessary to be between 18 and 65 years old, weigh at least 50 kg, and present a doctor’s certification. Every year a donor can donate up to 4 times for men and woman not of childbearing age and two times for women in the childbearing age, with a minimum interval of ninety days (Ministerial Decree 25/1/01). The maximum blood which can be donated at one time is 450 ml ± 10%. It is estimated that 40 units of blood are needed per year for every 1000 people, that is about 2,400,000 units only for Italy. In 2019 in Italy, 1,683,470 blood donors and among these 213,422 were donors in the younger group (18–25 years), and the new donors were just over 362,000, down by 2.3% [12].

However, it appears that there is a lack of donors compared to the actual needs. Therefore, it is a priority to investigate the propensity for donation among citizens to plan awareness actions and to identify the key factors and an effective incentive system to promote donation. On this strength, this study’s primary purpose is to understand the main determinants of citizens’ intention to donate blood and their propensity to encourage word of mouth about blood donation to identify similarities and differences between donors and non-donors.

To achieve this goal, we developed an empirical study in Italy to understand the main determinants of the individuals’ intention for donating blood [14–18].. We integrated the TPB considering further variables [19] on the differences between Italian donors and non-donors. A mixed-methods approach [20] was adopted.

The paper is structured as follows. Section 1 presents the literature review, the research hypotheses, and the conceptual model proposed. Section 2 offers the methodological approach, including the research plan, data collection and analysis. The results of our empirical study are presented in Section 3. Section 4 provides a discussion and findings. Finally, Section 5 provides conclusions, future perspectives and managerial implications.

## Main document

### Literature review and research hypotheses

Although there are many studies about blood donation, the majority of them were carried out in Anglo-Saxon countries [21]. There is a need to further investigate the phenomenon in other countries by developing empirical studies to analyse the main antecedents of citizens’ behavioural intention. The Theory of Planned Behaviour [14] has been primarily used to analyse blood donation intention, as it provides interesting insights for studying the phenomenon. Several researchers have reported that the TPB can be used to determine the predictors of blood donation [18, 22]. In particular, Reid and Wood [9] recognise the TPB as “the more appropriate model for investigating blood donation”. Hardeman et al. [23] emphasise the TPB contribution to the study of behavioural change interventions when the motivation to act is not known [22]. It is necessary to consider three main factors for studying the intention to donate by using the lens of the TPB: attitudes (overall evaluation of a specific behaviour), subjective norms (beliefs about the importance of others’ approval), and perceived behavioural control (beliefs about the ability undertake the proposed behaviour). Through the lens of the TPB in the blood donation context, it is possible to notice that donation behaviour can be affected by a positive attitude towards donation, a positive evaluation of donation among others and the perceived control of the donation experience [24].

However, France et al. [25] stated that additional factors might affect people’s motivations and behaviours regarding blood donation, emphasising the need for new studies on this research topic. Similarly, Reid and Wood [9] suggested considering a broader set of variables to increase this research’s usefulness. In line with this view, several authors [22, 24, 26–29] proposed extended and more comprehensive versions of the model to increase its predictive power [21]. For instance, Armitage and Conner [18] believe that the personal moral norm, namely, the sense of moral obligation, strongly impacts the intention to become a blood donor. Some authors [18, 22, 24, 30] that perceived behavioural control should be replaced by the construct of self-efficacy [31], that is, one’s perceived ability to perform the considered behaviour. To predict the intention to donate blood, Williams et al. [32] integrated the TPB with the self-determination theory (SDT) motivational variables proposed by Hagger and Chatzisarantis [33, 34]. Following the authors’ idea, this theoretical integration offers a complementary approach to identify the elements of blood donors’ behaviour, discovering that autonomous motivation has a positive direct effect on intention, as well as indirect effects via attitudes, subjective norms and perceived behavioural control. France et al. [35] introduced the “blood donation satisfaction” dimension as an antecedent of donor attitude; similarly, Schreiber et al. and Thomson et al. [36, 37] highlighted its influence on the retention of the donor’s status over time. The possibility of “helping other people” or “altruism” seems to be one of the most relevant motivations for both first-time and repeat donors to donate blood [6]. In light of the presented review, the TPB was adopted as the primary lens of our model to understand the determinants of blood donation, and we posit the following hypotheses:

H1: *Attitude* positively affects *intention to donate*.

H2: *Subjective norm* has a positive effect on *intention*.

H3: *Perceived Behavioural Control* has a positive effect on *intention*.

In literature, several authors call for a more in-depth and wider investigation to identify the elements that can motivate the citizens’, pushing them toward the blood donation [9, 25]. For instance, some authors [24, 38] have investigated the influence of donation knowledge on the intention to donate. It emerged that it is essential to create awareness around the donation, enhancing the dissemination of right and transparent information throughout an accurate communication. For instance, Williams et al. [32] suggested that developing “messaging designed to recognise and enhance an individual’s autonomy in deciding to donate again may be a more effective retention strategy than simply encouraging donors to return” [25]. Often, the lack of information on donating blood emerges as a reason for not donating [39]. WHO [10] states the role of communication is crucial to obtain the first donation and encourage first-time donors to return for repeat donations and generate a positive word of mouth (WOM). Indeed, communication is the core of a successful and sustainable blood donor program [10]. Effective communication strategies promote blood donation attitude [40]. For instance, the study conducted by Josefa et al. [41] highlights that the radiophone campaigns generate a change in the attitude to donate blood. Effective information and communication initiatives can then encourage people to change their behaviour by removing real or perceived inhibitors [41–44]. Communication strategies such as advertising, public relations, promotional campaigns, social media can play a crucial role in overcoming and mitigating these inhibitors to recruit and retain donors.

Moreover, Abbasi et al. [11] pointed out that social networks may play a crucial role in disseminating information, educating citizens, and sharing blood donation requests. In that sense, the role of traditional forms of communication should be strengthened by sharing information and experiences throughout individuals’ WOM and electronic WOM (e-WOM). Indeed, both WOM and e-WOM have a significant impact on consumer behaviour and decision-making [45] and a more significant influence on behaviour than other sources due to the reliability and flexibility of interpersonal communication and personal sources being viewed as more trustworthy [46, 47]. To understand the contribution of communication in fostering the blood donation, the following research hypotheses are formulated:

H4: *Communication* positively influences *attitude*.

H5: *Communication* positively influences *WOM*.

H6: *Communication* affects *Inhibitors.*

Multiple studies have investigated how donation-related fear and anxiety can negatively affect both the recruitment and retention of new donors. Indeed, the fear of donating blood is one of the main deterrents of becoming donors. As stated by France et al. [48], it directly affects donor retention rates and an indirect effect on increasing the risk of syncopal episodes [49].

Blood donation inhibitors are classified into physical risks (transmission of disease), psychological (fear), social (moral responsibility or religious aspects) also defined as internal inhibitors and lack of time, inconvenient schedule and location classified as external inhibitors [50, 51]. However, the most recurrent inhibitors that influence the intention to donate blood are fear of needles or fainting, the transmission of infectious diseases, pain when drawing and unpleasant sensations related to the withdrawal (fainting, weakness, nausea) [9, 52]. To better understand the causes of fear and anxiety, it is useful to identify ad hoc strategies to attract new donors and keep them for years. In that sense, Charbonneau et al. [53] advise investigating the obstacles together with donors’ demographic characteristics. Thus, the following research hypothesis is stated:

H7: *Inhibitors* influence *Intention*.

Sueming et al. [6] noticed that existing donors play an essential role in informing and motivating new volunteers. Martín-Santana and Beerli-Palacio [54] and Gazibara et al. [55] tested that donor experience is a factor influencing intention to donate blood in the future recommend donating blood to friends and relatives. Indeed, a donor’s positive experience encourages to re-donate, and the donor is more likely to generate Word of Mouth (WOM), therefore encouraging and promoting to donate blood [56]. On this base, we decided to test the relation between the intention of blood donation and WOM positing the following hypothesis:

H8: *Intention* affects *WOM.*

Finally, this study identifies the role of service quality in the blood donation process identifying the critical aspects of donor experience. It’s crucial to provide an optimal donation experience to promote and incentive blood donation [56, 57]. An effective and efficient donation system must consider and monitor service quality [55, 58] to ensure donor loyalty and satisfaction. If the donor has an awful experience generated by excessive waiting times to donate, impure structures, absence of support during and after the donation, medical staff unqualified [59] will decrease donors’ satisfaction and loyalty.

Anyway, there aren’t contributions that investigate the effects of service quality on the propensity to generate WOM in blood donation context and thus, we stated the following hypothesis to address this gap:

H9: *Service Quality has a positive effect on WOM.*

Starting from the above assumptions and theoretical background, we highlighted extensive literature on blood donation. Still, several authors have asked for further empirical studies to identify a larger number of antecedents related to the intention to donate. Moreover, only a few studies have discussed the similarities and differences between donors and non-donors [19]. In particular, the study conducted by Bednall et al. [21] emphasises that no previous studies have explored the effect of knowledge and awareness on donation behaviour, particularly taking into account and comparing donors versus non-donors. Similarly, a lack of research aimed to investigate the contribution of service quality in fostering blood donation.

On this strength, our study aims to understand the main antecedents of citizens’ intention to donate and the propensity to generate WOM, namely the propensity to recommend and communicate the value of blood donation and compare these factors donors and non-donors. We proposed a conceptual model grounded on the TPB model [14] and including other relevant variables for the blood donation and communication, inhibitors, service quality and WOM.

We want to emphasise that the same hypotheses were tested for both donors and non-donors. The main assumptions for the decision to analyse the two perspectives are following explained. It is vital to elicit the donors’ antecedents because they can provide insights for re-donate; on the other hand, it is crucial the perspectives of non-donors because it allows acting on overcoming some potential barriers to potentially donating and re-donate. Since the two groups present a different starting viewpoint, we expect some differences in the antecedents emerged; thus, we would like to highlight them.

**Fig. 1:**
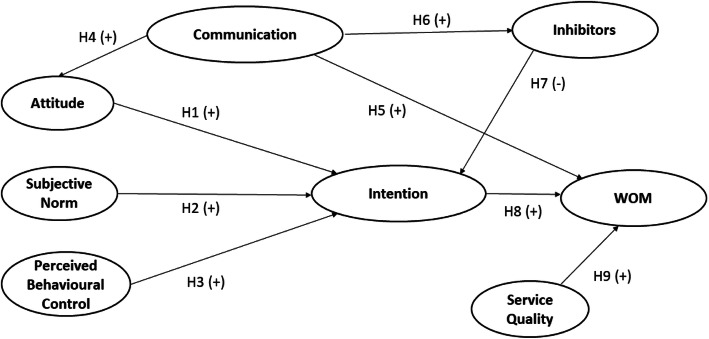
Conceptual model and research hypotheses

Figure 1 illustrates the hypotheses in the conceptual model.

## Methodology

An empirical study was conducted in Italy to understand better how to enhance citizens’ intention to donate and understand their propensity to donate. A mixed-methods approach was planned and implemented. As highlighted by several authors [60–63], the mixed methods approach combines qualitative and quantitative techniques to provide a more extensive and multifaceted analysis of a phenomenon. For this reason, in the last decade, the mixed methods approach has considered a methodological pillar [63, 64]. In particular, following the Priority-Sequence Model proposed by Morgan (1998) [20], a “qualitative preliminary approach” was adopted to guide the data collection in the principally quantitative step of the study.

Qualitative and quantitative surveys were conducted involving a sample of Italian citizens recruited in the country and did not receive any incentives for participating in our research.

The sample of the qualitative survey was composed of donors (*N* = 15) and non-donors (N = 15), while the quantitative survey sample was composed of donors (*N* = 173) and non-donors (*N* = 87).

### Research design, data collection, and analysis

The qualitative research explores the phenomenon of blood donation by investigating multiple aspects among both donors and non-donors. In general, the purpose of the semi-structured in-depth interviews was to examine and analyse the blood donation phenomenon’s strengths and weaknesses and compare them across both groups. Accordingly, the interview was carried out following two semi-structured topic guides one for the donors and one for the non-donor. The semi-structured interviews were composed of 7 guiding questions that were chosen a priori to facilitate discussion and maintain consistency [65], allowing respondents to express themselves naturally. Additional aspects were explored when raised by the respondents [65]. The semi-structured in-depth interview guides (Appendix I) were structured as follows: first, general questions on the phenomenon were used for both groups, and then, some customised questions were posed based on the “status” of the respondent (donors/non-donors). In the donors’ case, the motivations for the experience of donations were investigated, whereas obstacles and shortcomings were examined in depth for the non-donors. In both cases, suggestions to increase the propensity towards blood donation were assessed.

After they donated at the blood transfusion centre of an Italian hospital located in Rome (Italy), the donors’ sample was selected. The non-donors were selected through a snowball approach [66]. In February 2018, by adopting the themes saturation criteria [67], 30 individuals underwent face-to-face in-depth interviews (15 = donors; 15 = no-donors). The interviews, approximately 30/40 min each, were audio-recorded, transcribed in verbatim and subjected to hermeneutical and analysed by content analysis [68]. Specifically, to analyse the qualitative data, we followed the four phases of content analysis: coding, categorising, thematising and integrating [68–70]. The MAXQDA18 software was adopted to manage and analyse the data, and we followed a rigorous process to reduce any potential research bias. First, the collected qualitative data were coded in parallel by two researchers; a third researcher performed a second comparison of the two results. Finally, the discussion and the interpretation of the content analysis were jointly performed by the three researchers.

Thereafter, integrating the literature review and the qualitative results, a quantitative analysis was planned to investigate the attitudes, motivations and behaviour of both donors and non-donors. Hence, two questionnaires were developed based on the TPB, previous studies on the same topic [19], and the qualitative analysis findings. The two questionnaires were structured in the same way; only three items slightly differ due to the specific status of donors and non-donors. Hence, eight common dimensions were defined: *Attitude*, *Subjective norm*, *Perceived behavioural control*, *Inhibitors and obstacles to donat*ing, *Information and Communication*, S*ervice quality*, *Intention*, *Word Of Mouth*. Both questionnaires were composed of 29 items. A seven-point Likert scale was adopted to gather responses (1=“ completely disagree” to 7=“ completely agree”). The dimensions and items proposed in the questionnaires are shown in Table 1.

**Table 1:** Questionnaire structure for Donor and Non-Donors (in brackets the item’s text adapted for Non-Donors group)

Dimension	Item	Code	References
*Attitude*	I think that donating blood is ethical.	ATT_1	[11]
	I think that donating blood is useful.	ATT_2	
	I think that donating blood is safe.	ATT_3	
	I think that donating blood is a moral obligation.	ATT_4	
	Donating blood is important to me.	ATT_5	
	I think that donating blood is a personal responsibility.	ATT_6	
*Subjective norm*	Most people that are important to me appreciate that I am a donor. [most people that are important to me would appreciate if I became a donor]	SN_1	[11]
	Most people that are important to me think that I should donate blood.	SN_2	
	Most people that are important to me appreciate the donation activity.	SN_3	
*Perceived behavioural control*	It is easy to possess the requisites to donate.	PBC_1	[11]
	People who have a regular life are more likely to be blood donors.	PBC_2	
	If I decide to donate in the next weeks, I could do it without difficulty.	PBC_3	
*Inhibitors*	Fear of needles.	INHI_2	[16] Qualitative phase
	Pain when drawing.	INHI_3	
	Sight of blood.	INHI_4	
	Unpleasant sensations related to the withdrawal (fainting. Weakness. nausea).	INHI_5	
	Withdrawal preparation (compliance with requirements).	INHI_6	
*Information and communication*	It is necessary to increase donation awareness activities.	COM_1	Qualitative phase
	It is necessary to make young people aware of donation through activities in schools and universities.	COM_2	
	It is necessary to increase donation awareness through social media and social networking.	COM_3	
	It is necessary to introduce promotional campaigns through web and social networks	COM_4	
*Service quality*	The medical staff should be kind.	SQ_2	[32, 65, 66]
	The medical staff should be competent.	SQ_3	
	The medical staff should support me during and after the donation.	SQ_5	
*Intention*	I would like to donate blood again [I would like to become a donor]	INT_1	[11, 16]
	I would like to donate blood more often [I will donate blood for the first time]	INT_2	
	I would like to donate blood even without receiving benefits (e.g., discounts, economic benefits).	INT_3	
WOM	I would recommend blood donation to my friends and family.	WOM_1	[11, 16]
	I would recommend blood donation on social networks.	WOM_2	

Both questionnaires close with an open question, aimed at allowing participants to explain the main motivations behind their behaviour to donate/do not donate. These two open questions were analysed by classifying and coding the motivations shared by the respondents.

The questionnaire was tested through a pilot survey on a sample of 30 respondents. The formulations of some questions were adapted to improve the clarity and consistency of items and dimensions. Then, the quantitative survey was administered via the web using the support of social networks and the institutional websites of blood donation associations and foundations (October–November 2018). The convenience sample used in this study was considered appropriate for addressing the aim of the research [71, 72]. The sample size is suitable for testing the statistical significance of the hypothesised relationships for both the groups of donors and non-donors.

Here, the semantic meaning of the proposed dimensions is explained.

“Attitude” towards blood donation assesses whether a respondent believes that this activity is ethical, safe, useful, and a citizen’s moral and social obligation. “Subjective norm” considers beliefs about whether significant other people approve of and appreciate blood donation behaviour. Indeed, the construct is generated by the perception that other people appreciate blood donation and the recurrence of donating. “Perceived Behavioural Control” indicates the degree to which people think they can control a specific behaviour such as having the requisites and a lifestyle suitable for donating and not find it difficult to donate. “Information and Communication” assesses citizens’ perceptions of the need to increase donation awareness through mass media, promotional campaigns on social networks and educational initiatives in schools/universities.

“Service quality” assesses the perceptions attributed to the kindness, competence and availability of medical staff. “Inhibitors and obstacles to donating” assess some unpleasant sensations related to blood donation and personal fears related to blood donation (i.e., fainting, fear of the needle, the sight of blood, pain). “Intention” assesses the willingness to donate in the future (or for the first time) and more often but without receiving benefits (discounts, economic benefits, etc.) as the Italian regulation foresees it. “Propensity to Generate WOM”, is namely the propensity to recommend and communicate the value of blood donation to other people. Thus, this dimension assesses respondents’ intention to recommend blood donation to friends and family face-to-face and on social media and social networks.

According to data analysis, firstly, the reliability and validity of the multi-item scales were verified according to the internal consistency (Cronbach’s alpha) and the convergent validity through Average Variance Extracted (AVE) and Composite Reliability (CR) [73, 74].

Then, the data analysis was carried out using the SPSS IBM 17.0 and Mplus 7 software packages [75]. Structural equation modelling (SEM) was used to verify the relations and test the conceptual model [76]. In particular, the multi-group SEM allows to simultaneously test the same model on multiple independent samples, based on the possession of a specific characteristic (donors, non-donors) [67].

## Results

This section presents the qualitative survey results (4.1) and the quantitative survey (4.2).

### Qualitative results

During the preliminary qualitative phase of the analysis, 30 in-depth interviews were conducted (15 = donors; 15 = non-donors). The sample’s composition was balanced for pursuing explanatory power concerning different characteristics of the two distinct groups.

As shown in Appendix II, the sample of donor interviewees is composed of 7 males and 8 females, and the age range is balanced as follows: 18–25 (2); 26–35 (6); 36–45 (6); 46–55 (1). The majority of interviewees are regular donors (9); they donate 3–4 times a year. While the non-donors interviewed are 5 males and 10 females. The highest number of non-donors is found in the age groups: 18–25 (6); 26–35 (7); 36–45 (2).

During the analysis, the divergences of subjective interpretation and codification were discussed or reanalysed to solve the conflicting view [77]. The results were compared to identify the vital common aspects and priorities for both donors and non-donors.

**Fig. 2:**
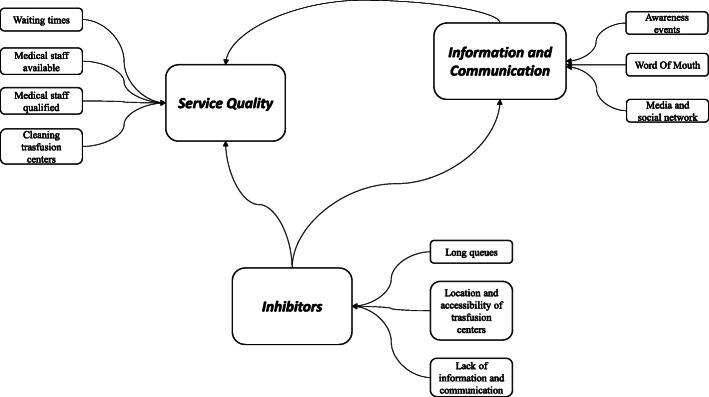
Qualitative results: the donors’ cognitive map

**Fig. 3:**
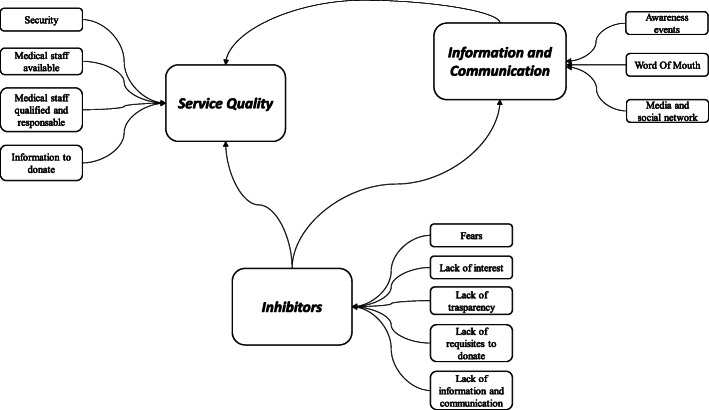
Qualitative results: the non-donors’ cognitive map

The qualitative analysis’s main output has been summarised by developing a cognitive map for donors and non-donors (Figs. 2, 3) and a table explaining donation meaning and motivations (Table 2).

**Table 2:** Qualitative results: frequency of recurrent key issues for donors and non-donors

Donors		Non-donors	
The donation is …	Frequency	The donation is …	Frequency
a personal responsibility	11	… a personal responsibility	10
a form of altruism	9	… a form of altruism	9
a moral obligation	6	… a moral obligation	3
**The donor is a person …**	**Frequency**	**The donor is a person …**	**Frequency**
with a healthy lifestyle	7	...fear	13
altruistic	6	...with a healthy lifestyle	6
responsible	2	...altruistic	4
		...responsible	3
		...courageous	2
**Motivations to donate**	**Frequency**	**Motivations not to donate**	**Frequency**
personal values	7	Fear	8
to help friends/family members	6	Requisites to donate	3
external influences	2	Transparency	2
		Interest	2

The maps showed the three main cross dimensions from the qualitative interview analysis: i) Service quality, ii) Information and Communication and iii) Inhibitors to donation.

The main difference that emerged was related to the motivations at the basis of the process of donation. In particular, the Inhibitors play an important role for the non-donors, because they represent the intimate obstacles of individuals such as fear of the blood or fear for lack of safety etc.

The content analysis revealed that service quality aspects are pivotal for individuals engaged in the blood donation process. In particular, the donor respondents take into account waiting times to donate, the cleanliness of transfusion centres and the availability and professionalism of the medical staff (Fig. 2) (i.e. *“When I donate I pay attention to whether medical staff are friendly and qualified, polite treatment and to tangible aspects such as the cleanliness of the facilities”).* Moreover, the non-donors considered the security of transfusion centres and easy access to information about donation (e.g., places and times) as strengths of service quality (i.e. *“The transfusion centres must guarantee the easy access to the donation centre and the easy-to-find information about places and times. Transfusion centres must also be safe and therefore guarantee hygiene and staff qualified”).*

Indeed, the qualitative analysis shows that the low propensity to donate among non-donors is justified by intimate psychological factors (i.e., needles, infectious diseases, the sight of blood), the physical characteristics that inhibit donation (e.g., low blood pressure and abnormal blood levels), (i.e. *“I can’t donate due to my health condition.” “The sight of blood is unpleasant and I’m afraid of needles and of infectious disease transmission”),* the lack of communication and information about initiatives, the lack of interest and the lack of transparency in the system, which generates insecurity. Respondents argued that there is a low propensity to donate among young people due to the lack of information, disinterest and a loss of moral values. (i.e. “*It is necessary to meet young people and take initiatives in schools and universities to sensitise them to blood donation”).* For donors, the main obstacles to donation are long queues, the location and accessibility of transfusion centres as well as lack of information and communication about blood donation events and initiatives. (i.e. *“In small towns, people are not informed about the importance of donation. Donation initiatives are not advertised. Information is often not provided on the places, days and times to donate.”).* Both donors and non-donors suggested promoting communication-related to blood donation events by using traditional WOM and advertising campaigns on social networks (e-WOM) and educational events in schools and universities. (i.e. *“More communication and involvement in the donation are needed. Social media platforms and influencers should be used to receive and transmit information on blood donation campaigns and requests.”. “Given the lack of blood, the advertising campaign should be increased, especially in the summer, given the low number of donors.”).*

As shown below (Table 2), both groups consider blood donation a personal responsibility (21) and a custom of altruism and generosity (18) that creates collective well-being. For donors, the donation is a moral obligation (6). Donors believe in the intrinsic values of donation (7); they donate to help friends/family (6) or for external influences (2) such as meeting new people, having a free check-up or obtaining social recognition among friends/family. The main motivations for not donating are fear (8), which includes fear of needles, the sight of blood, bruising and adverse reactions or the lack of requisites to donate (3). Besides, the non-donors do not donate due to the lack of transparency (2), which generates insecurity or is not interested in blood donation (2). Donors are perceived as people with a healthy lifestyle (20), people who are altruistic (12) and people who are responsible (7). Also, the non-donors perceive donors as courageous (3) and religious (2).

### Quantitative results

The sample comprises 260 respondents, divided into donors (*N* = 173) and non-donors (*N* = 87). Next, the results of the collected data from the two questionnaires are shown.

#### Sample description

An overview of the sample characteristics is shown in Table 3.

**Table 3:** Demographic characteristics of the quantitative samples

Demographic characteristics		Sample’s specifics			
		***Donors (N = 173)***		***Non-donors (N = 87)***	
		*Frequency*	*Percentage*	*Frequency*	*Percentage*
*Gender*	Male	87	50.3%	30	34.5%
	Female	86	49.7%	57	65.5%
*Age*	18–24	30	17.3%	20	23.0%
	25–34	67	38.7%	46	52.9%
	35–44	32	18.5%	12	13.8%
	45–54	33	19.1%	5	5.7%
	55–64	10	5.8%	2	2.3%
	> 65	1	0.6%	2	2.3%
*Educational level*	Elementary school	17	9.8%	/	/
	High school	104	60.1%	30	34.5%
	Bachelor’s	19	11.0%	32	36.8%
	Master’s	25	14.5%	19	21.8%
	MBA	7	4.0%	5	5.7%
	PhD	1	0.6%	1	1.1%
*Job*	Civil servant	99	57.2%	38	43.7%
	Private sector employee	13	7.5%	8	9.2%
	Student	42	24.3%	30	34.5%
	Unemployed	17	9.8%	9	10.3%
	Retiree	2	1.2%	2	2.3%

The sample of donors is composed of 173 respondents, including 87 males (50.3%) and 86 females (49.7%) who belonged to the 18–24 (17.3%), 25–34 (38.7%), 35–44 (18.5%), 45–54 (19.1%), 55–64 (5.8%) and over 65 (0.6%) age ranges. High school is the most common level of education (60.1%) of donors. A total of 57.2% of donors are civil servants, and 24.3% are students.

Regarding the donation career of respondents emerged that:

- 33 donors (10 males, 23 females) donate occasionally once a year;

- 53 respondents (12 males, 41 females) donate blood two times a year;

- 38 respondents (28 males, ten females) donate three times a year;

- 49 donors (37 males, 12 females) are regular donors (4 times a year).

The non-donors sample included 87 respondents, of which 65.5% were females, and 34.5% were males. The majority of the non-donor sample (52.9%) is in the 25–34 age range. The other respondents belonged to the following age ranges: 18–24 (23%), 35–44 (13.8%), 45–54 (5.7%), 55–64 (2.3%) and over 65 (2.3%). The majority of the sample had a bachelor’s degree (36.8%); 43.7% were civil servants, 34.5% were students, and 10.3% were unemployed.

The distribution of the respondents across Italian regions (Appendix III) shows that the significant number of respondents are Veneto (25.4%), Piedmont (17.9), Latium (17.9%) and Puglia (11.6).

Concerning the motivations behind the donation, from the analysis of the open questions, it emerged that the main aspects enticing donors to donate are the following: personal choice and beliefs (37%), educational activities (17%), to have accompanied relatives and friends to donate (13%), and sensitive campaigns (13%) (Table 4). In contrast, donating’s favourite locations seem to be schools and universities (45%) and ad hoc areas in the city centre (24%). For non-donors, the primary aspect that may encourage them to start donation dating is the needs of blood from friends and family members (54%) followed by sensitive companies (21%) (Table 4).

**Table 4:** Quantitative results: frequency of donor’s motivations to blood donation and a favourite location for donating (a) and frequency of motivations that could push Non-Donors to donate and favourite location to start donating (b)

DONORS (a)					
Motivation for first donation	Frequency	%	Favourite location for donating blood	Frequency	%
Individual choice	64	37%	School/universities	77	45%
Educational initiatives	29	17%	City centre areas (plaza or parks)	42	24%
Accompanying relatives/friends	23	13%	In the office	24	14%
Sensitive campaign	23	13%	Sport centre	12	7%
Blood need for relatives/friends	14	8%	Hospitals	7	4%
Being a parent of a donor	8	5%	Blood donation centre	2	1%
Altruism	6	3%	Churches	2	1%
Being a friend of a donor	2	1%	Everywhere	2	1%
Free breakfast	1	1%	Place with parking	2	1%
Familiar education	1	1%	Associations	1	1%
Personal motivation	1	1%	Mall	1	1%
Personal satisfaction	1	1%	Game rooms	1	1%
**NON-DONORS (b)**					
**Motivations for starting the donation**	**Frequency**	**%**	**Favourite location to start donating**	**Frequency**	**%**
My parents/friend need blood	47	54%	Doesn’t matter	45	52%
Sensitive campaigns	18	21%	Blood donation centre	34	39%
Supporting those who are in need	9	10%	Association centre	5	6%
For ethical reasons	6	7%	Mobile blood station	3	3%
Educational event at school/university	3	3%			
Accompanying parents/friends	3	3%			
Overcoming my fears	1	1%			

#### Donors’ and non-donors’ internal reliability and validity

Regarding the donor dataset, the internal reliability of each factor was calculated by using Cronbach’s alpha coefficient [78], and the construct validity Convergent Variance Extracted (AVE) and Composite Reliability (CR). All the data meet the criteria for acceptable reliability and validity: 0.7 for Cronbach’s alpha [73, 79, 80], 0.5 for AVE and 0.7 for CR [74]. Additionally, for the non-donor dataset, reliability and validity were calculated using the same measures.

As shown in Table 5, the data meet the criteria for acceptable reliability and validity [73, 79, 80].

**Table 5:** Constructs reliability and validity: Cronbach’s alpha, Average Variance Extracted (AVE) and Composite Reliability (CR) for Donors (Group A) and Non-donors (Group B)

Group A: DONORS				Group B: NON-DONORS			
Factor	Cronbach’s alpha	AVE	CR	Factor	Cronbach’s Alpha	AVE	CR
***Attitude***	0.910	0.638	0.913	***Attitude***	0.880	0.562	0.884
***Subjective norm***	0.872	0.695	0.871	***Subjective norm***	0.804	0.547	0.783
***Perceived behavioural control***	0.842	0.619	0.828	***Perceived behavioural control***	0.750	0.584	0.806
***Inhibitors***	0.897	0.639	0.898	***Inhibitors***	0.840	0.575	0.868
***Information and Communication***	0.941	0.759	0.926	***Information and Communication***	0.900	0.671	0.889
***Service Quality***	0.934	0.816	0.930	***Service Quality***	0.900	0.734	0.892
***Intention***	0.777	0.553	0.784	***Intention***	0.753	0.625	0.826
***WOM***	0.891	0.821	0.901	***WOM***	0.841	0.731	0.844

#### Structural equation models: a multi-group analysis

The conceptual model was tested with SEM using *Mplus* 7 software [75].

The adopted procedure is as follows. First, we separately developed models for Group A, i.e., the donors (*N* = 173), and Group B, i.e., the non-donors (*N* = 87). Then, we used the multi-group analysis to identify the main differences between the two independent samples simultaneously. The invariance between the two samples was tested by using multi-group SEM. The baseline model was fitted to the data on both groups simultaneously, χ2 (df = 715) = 1.326.504, *p* < .01, CFI = .903, RMSEA = .080 (95% CI = 0.074 0.088), SRMR = .065, supporting the configural invariance hypothesis. Then, constraining the loadings between the groups yielded a nonsignificant increase of the CFI (ΔCFI = .003), providing support for metric invariance. Moreover, constraining the intercepts between the groups, we observed a small decrease in the CFI: (ΔCFI = .003). The model is assumed to be non-invariant if the decrease in CFI is larger than 0.002 [81] compared to the baseline model. We have not considered the difference between the chi-square of nested models considering the strong dependence of the chi-square on the sample size [82]. Thus, the hypothesis of scalar invariance can be accepted.

Hence, a graphical representation of the model is proposed. The robust estimator MLMV was used for continuous variables to correct covariance. Table 6 shows the results of the goodness-of-fit parameters. Then, a graphical representation of the measurement models is proposed for both groups.

**Table 6:** Goodness-of-fit index model for Donors (Group A) and Non-donors (Group B)

Goodness-of-fit index	Observed value	Commonly used threshold
Χ^2^ (Chi-squared)	934.259*	[55, 58–60]
degrees of freedom	760	
p-value	0.000	
Χ^2^ (Chi-squared) contribution group A	481.191	[67]
Χ^2^ (Chi-squared) contribution group B	453.068	
SRMR (Standardized root mean square residual)	0.074	< 0.08 [60]
CFI (Comparative fit index)	0.907	≥ 0.90 [57]
TLI (Tucker-Lewis index)	0.901	≥ 0.90 [58]
RMSEA		< 0.05: minimal error
Root mean square error of approximation	0.042	0.05 ≤ RMSEA ≤0.08 acceptable
90% C.I. = (0.032–0.051)		≥ 0.08 rejectable model [56]
WRMR (Weighted root mean square residual)	0.985	< 1 [69]

The results of the SEM goodness-of-fit parameters are presented below (Table 6):

- *Root mean square error of approximation* (RMSEA = 0.073; 90% C.I. = 0.066;0.080): acceptable according to Browne and Cudeck [83];
- *Critical fit index* (CFI = 0.915): acceptable according to Bentler [84];
- *Tucker-Lewis index* (TLI =0.901): acceptable according to Tanaka [85];
- *Standardised root mean square residual (*SRMR = 0.062): acceptable according to Hu and Bentler [86].

The analysis confirms that the χ^2^ (chi-squared) value is significant with its linked probability value. The χ2 test was statistically significant, which indicates an unsuitable fit, even if, according to several authors, it needs to be compared with other indexes before rejection [85–88].

**Fig. 4:**
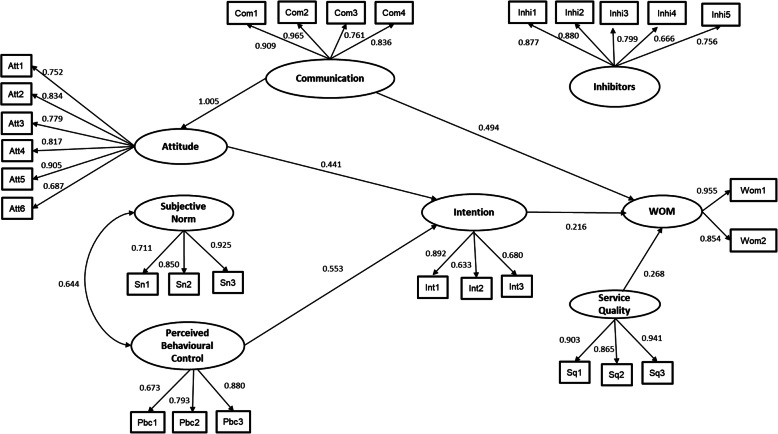
Quantitative results: The structural model of Group A (Donors). The structural equation model of Group B (Non-donors) for the prediction of blood donation determinants and WOM

**Fig. 5:**
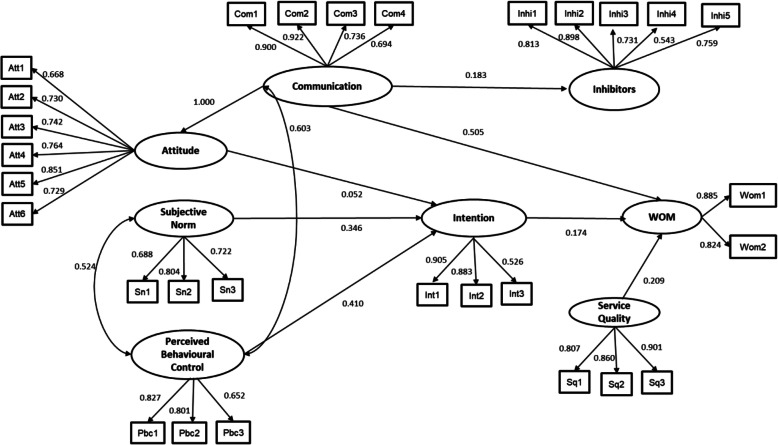
Quantitative results: The structural equation model of Group B (Non-donors) for the prediction of blood donation determinants and WOM

The other indicators of goodness of fit can be considered adequate since all the values fall within the thresholds suggested by the literature. The graphical representation of the model is shown for both groups: donors (Fig. 4) and non-donors (Fig. 5), including only the significant relations between factors (*p* <  0.05).

Group A’s observed model shows that there is the covariance between Subjective Norm and Perceived Behavioural Control (β = 0.644), as in the model previously tested by Ajzen [14].

The observed model of Group B shows that there is the covariance between Subjective Norm and Perceived Behavioural Control (β = 0.603), as in the model previously tested by Ajzen [14], as well as between Perceived Behavioural Control and Communication (β = 0.524).

The results of the two groups are summarised in Tables 7. It is possible to notice that the indicators have significant loadings on their assigned constructs. The residual variances are reported in Appendix IV.

**Table 7:** Factor loadings statistics, Donors (Group A) and Non-donors (Group B)

Constructs	Group A: DONORS				Group B: NON-DONORS			
	Code	Standardized loading	Measurement error variance	P-value	Code	Standardized loading	Measurement error variance	P-value
Attitude	ATT_1	0.752	0.048	0.000	ATT_1	0.668	0.036	0.000
	ATT_2	0.834	0.035	0.000	ATT_2	0.730	0.032	0.000
	ATT_3	0.779	0.045	0.000	ATT_3	0.742	0.033	0.000
	ATT_4	0.817	0.038	0.000	ATT_4	0.764	0.038	0.000
	ATT_5	0.905	0.023	0.000	ATT_5	0.851	0.024	0.000
	ATT_6	0.687	0.043	0.000	ATT_6	0.729	0.032	0.000
Subjective Norm	SN_1	0.711	0.041	0.000	SN_1	0.688	0.039	0.000
	SN_2	0.850	0.036	0.000	SN_2	0.804	0.041	0.000
	SN_3	0.925	0.018	0.000	SN_3	0.722	0.039	0.000
Perceived Behavioural Control	PBC_1	0.673	0.043	0.000	PBC_1	0.827	0.028	0.000
	PBC_2	0.793	0.032	0.000	PBC_2	0.801	0.036	0.000
	PBC_3	0.880	0.030	0.000	PBC_3	0.652	0.034	0.000
Inhibitors	INHI_1	0.877	0–025	0.000	INHI_1	0.813	0.034	0.000
	INHI_2	0.880	0.024	0.000	INHI_2	0.898	0.023	0.000
	INHI_3	0.799	0.028	0.000	INHI_3	0.731	0.034	0.000
	INHI_4	0.666	0.045	0.000	INHI_4	0.543	0.045	0.000
	INHI_5	0.756	0.032	0.000	INHI_5	0.759	0.039	0.000
Information and Communication	COM_1	0.909	0.019	0.000	COM_1	0.900	0.022	0.000
	COM_2	0.965	0.011	0.000	COM_2	0.922	0.020	0.000
	COM_3	0.761	0.035	0.000	COM_3	0.736	0.041	0.000
	COM_4	0.836	0.030	0.000	COM_4	0.694	0.040	0.000
Service Quality	SQ_1	0.903	0.019	0.000	SQ_1	0.807	0.028	0.000
	SQ_2	0.865	0.041	0.000	SQ_2	0.860	0.021	0.000
	SQ_3	0.941	0.016	0.000	SQ_3	0.901	0.023	0.000
Intention	INT_1	0.892	0.026	0.000	INT_1	0.905	0.028	0.000
	INT_2	0.633	0.046	0.000	INT_2	0.883	0.018	0.000
	INT_3	0.680	0.071	0.000	INT_3	0.526	0.041	0.000
WOM	WOM_1	0.955	0.015	0.000	WOM_1	0.885	0.032	0.000
	WOM_2	0.854	0.041	0.000	WOM_2	0.824	0.028	0.000

The main results and the status of the research hypotheses for both groups are summarised in Table 8.

**Table 8:** Status of research hypotheses for Donors (Group A) and Non-donors (Group B)

	Hypothesis	Predictor	Dependent variable	Estimate	S.E.	Two-tailed p-value	Supported
**Group A** **DONORS**	**H1**	*Attitude*	*Intention*	0.441	0.136	0.001	Yes
	**H2**	*Subjective Norm*	*Intention*	−0.031	0.083	0.705	No
	**H3**	*Perceived Behavioural Control*	*Intention*	0.553	0.121	0.000	Yes
	**H4**	*Communication*	*Attitude*	1.005	0.052	0.000	Yes
	**H5**	*Communication*	*WOM*	0.494	0.076	0.000	Yes
	**H6**	*Communication*	*Inhibitors*	−0.066	0.060	0.271	No
	**H7**	*Inhibitors*	*Intention*	−0.025	0.039	0.517	No
	**H8**	*Intention*	*WOM*	0.216	0.069	0.002	Yes
	**H9**	*Service Quality*	*WOM*	0.268	0.079	0.001	Yes
**Group B** **NON-DONORS**	**H1**	*Attitude*	*Intention*	0.052	0.078	0.054	Yes
	**H2**	*Subjective Norm*	*Intention*	0.346	0.106	0.001	Yes
	**H3**	*Perceived Behavioural Control*	*Intention*	0.410	0.081	0.000	Yes
	**H4**	*Communication*	*Attitude*	1.000	0.097	0.000	Yes
	**H5**	*Communication*	*WOM*	0.505	0.088	0.000	Yes
	**H6**	*Communication*	*Inhibitors*	0.183	0.062	0.003	Yes
	**H7**	*Inhibitors*	*Intention*	−0.039	0.057	0.493	No
	**H8**	*Intention*	*WOM*	0.174	0.066	0.009	Yes
	**H9**	*Service Quality*	*WOM*	0.209	0.107	0.052	Yes

Regarding the donors, all the proposed hypotheses are supported (*p*-value < 0.005), except for H2, H6 and H7. The observed model in Group A (donors) shows that Attitude (β = 0.441) and Perceived Behavioural Control (β = 0.553) directly and positively influence Intention (H1, H3). Communication has a substantial impact on attitude (β = 1.005) and on Propensity to Generate WOM (β = 0.494) (H4, H5). The propensity to Generate WOM is influenced by intention (β = 0.216), Service Quality (β = 0.268) and Communication (β = 0.494) (H8, H9, H5). However, Subjective Norm (β = − 0.031) and Inhibitors (β = − 0.025) do not significantly affect intention to donate (H2, H7), and communication (β = − 0.066) does not significantly affect Inhibitors (H6) (*p* > 0.05). In particular, Subjective Norm does not affect Intention or Inhibitors. Besides, communication does not influence Inhibitors (β = 0.271).

Regarding the non-donors, all the proposed hypotheses are supported (*p*-value < 0.05), except H1 and H7. In particular, the results reveal that Subjective Norms (β = 0.346), Perceived Behavioural Control (β = 0.410) and Attitude (β = 0.052) affect intention to donate (H2, H3, H1). Concerning the construct Attitude, its p-value can be considered marginally significant (p-value = 0.054), and for the principle of conservation, we decided to accept H1. Communication positively influences attitude (β = 1.000) and Inhibitors (β = 0.183) (H4, H6). Communication (β = 0.505), Intention (β = 0.174) and Service Quality (β = 0.209) affect Propensity to Generate WOM (H5, H8, H9). However, Inhibitors (β = − 0.039) does not affect the intention to donate (p-value = 0.493) (H7). In particular, Inhibitors is not a significant antecedent of the intention to donate among non-donor respondents.

Summarising, the donors group’s results show that Attitude and Perceived Behavioural Control are antecedents of intention to donate (again). Subjective Norm does not affect the intention. Moreover, the results reveal that Inhibitors influence neither intention to donate nor the WOM, which makes sense in donors’ case. Communication and information, which has no impact on Inhibitors, affect Attitude and Propensity to Generate WOM, affected by Intention and Service Quality.

While, the case of non-donor Attitude, Subjective norm and Perceived Behavioural Control directly influence the intention to donate (for the first time). Even among non-donors, Information and Communication predict both Attitude and Inhibitors. Regarding propensity to generate WOM, there are three main predictors: Intention, Service Quality and Communication. The non-donors’ propensity to generate WOM is affected by their intention and their importance to Service Quality. Among non-donors, attitude is even influenced by communication, and communication has a positive impact on Inhibitors and Propensity to Generate WOM.

## Discussion and findings

The present paper proposes a conceptual model grounded on the literature and aimed to study the main determinants of the donation intention and the propensity to empower the donation WOM. Throughout adopting the mixed-method approach, qualitative and quantitative research was integrated to verify the model on two different groups: donors and non-donors.

The qualitative step of the research confirms the existence of multiple dimensions affecting the decision to donate for both groups, highlighting the importance to propose more complex model respect to the TPB [14] including further variables as suggested by other authors [9, 25]. More specifically, the qualitative results confirm the importance of service quality to repeat and promote the donation [44, 57]. Similarly, the qualitative analysis corroborates the presence of a long list of perceived inhibitors that hamper the donation and identified in the literature analysis [e.i. 41,43,48,49,50,51,53,58,59]. Finally, it seems to emphasise the central role of the information and communication processes in fostering both the retention and promotion of blood donation [e.i. 10,25,40,46,47]. These qualitative findings were used as input to develop the quantitative questionnaires aimed to test the proposed conceptual model. Indeed, the model was simultaneously tested on two independent samples of donors and non-donors, and the results are very interesting. The goodness of fit indexes can be considered adequate following the literature thresholds. Thus, the conceptual model is validated by our data. Some differences and similarities in the antecedents of blood donation are found between the two groups.

It is worth emphasising that our findings confirmed the relations identified by previous studies [14], namely, Attitude and Perceived Behavioural Control are predictors of intention for donors. In contrast, Attitude, Subjective Norm and Perceived Behavioural Control are the main predictors for non-donors. Unlike non-donors, donors decide to donate blood regardless of social influences. While, for non-donors, social influences affect the decision to donate, extending beyond the individual’s appraisals on blood donation and the perceptions of how difficult or easy it will be performing the donation.

These findings are aligned with previous studies that have implemented the TPB model for blood donation [18, 22]. Nevertheless, some new constructs were included in the present study to fill the gaps in knowledge identified in the literature review [e.i. 9, 25].

Indeed, the present study suggests that *Service Quality* is an essential dimension for both donors and non-donors. Assessments of Service Quality include the individual’s perceptions of the kindness, competence and availability of medical staff, waiting times for donation, cleanliness of transfusion centres and ease of finding information on places and times where donation occurs. It is vital to enhance the propensity to generate WOM among donors. Indeed, donation centres need to improve the quality of their services to be more attractive to donors. The medical staff must be kind, available and organised to reduce waiting times. Donation centres should be clean and provide information on days and places where people can donate, preferably giving appointments to donors via the web. This is aligned with the hypothesis of Pagliariccio and Marinozzi [89] concerning the positive influence of donation satisfaction on the behaviour to donate again.

Moreover, our findings recognise that the construct *Information and Communication* is crucial for both donors and non-donors, indicating a need to sensitise and increase donation awareness through mass and social media by developing recruitment campaigns mainly on social networks and through promoting educational activities in schools and universities. Communication should promote donations mostly among young adults, provide clear and educative information, explain the process of donation and the concrete experience, describe legal health requirements, and ensure citizens that the donation process is safe. This centralised mass and virtual communication process could have a positive impact not only in engaging new donors among young people and millennials but also in recruiting previous donors.

Finally, *Inhibitors* that represent the unpleasant sensations related to the blood draw and personal fears related to the blood draw (i.e., fainting, fear of the needle, the sight of blood, pain) were not a significant direct predictor of intention in either group. Our results confirmed that the donors’ awareness of the importance to donate prevails over inhibitors for the donors’ sample. For the non-donors, a crucial role is played by the information and communication initiatives that can mitigate real and perceived inhibitors by encouraging people to recommend to friends/family and on social networks to donate.

Anyway, both groups are affected by Communication and Information that influence the Propensity to WOM. People who are inclined to Inhibitors are more difficult to be recruited and sensitised to blood donation. Although non-donors cannot be easily converted into donors because they cannot overcome those obstacles [90–92], information and communication could sensitise them to blood donation and overcome their limits.

Although they are non-donors, they can generate WOM for blood donation, by becoming vehicles of promotion. Moreover, non-donors are also susceptible to Subjective Norm that considers beliefs about whether significant other people approve of and appreciate blood donation behaviour. Even if they are not donors (for instance, for lack of requirements or fear), they may promote the importance of donating blood within their networks, particularly among those who have the specific features to become donors and, at the same time.

## Conclusion

### Originality and managerial implications

From an academic viewpoint, our study’s originality stands on the analysis of the blood donation phenomenon to understand the antecedents of citizens’ intention to donate and their propensity to recommend and communicate the value of blood donation. Also, the research proposes a combined analysis of two different groups: donors and non-donors.

Our findings show differences and similarities in the antecedents of blood donation among donor and non-donor groups. The study confirms the TPB’s appropriateness in analysing the blood donations phenomenon, introducing further relevant dimensions that have an important role as a determinant to donate and promote a positive WOM towards the donation. These dimensions are Attitude, Perceived Behavioural Control, Information and Communication, Service quality, Intention and WOM.

Our findings can also provide useful insights at different levels (macro, meso and micro) to promote blood donation.

At the macro level, including the government and policymakers such as the Health Ministry, our study highlights the vital role of information and communication for developing effective strategies to promote blood donations in the Italian community. Moreover, social media and networks can play a fundamental role in promoting blood donation activity through educational activities.

The macro-level role is crucial for proposing effective and efficient strategies able to promote blood donation as much as possible, orienting the meso level to take concrete actions to educate citizens, especially young people and millennials. The meso level is represented by companies, healthcare organisations, schools and universities. In the era of smart working and e-learning, the realisation and administration of educational videos and e-seminars on blood donation should be worthwhile for converting non-donors into donors and to reinvigorate previous donors. Moreover, a good campaign of communication could also be useful for acting on non-donors Inhibitors. The information and communication should be clear and detailed regarding the procedures and the safety of the process, guaranteeing safeguard and protections to donors. Furthermore, our study suggests to healthcare organisations and blood associations that the Service Quality of blood centres may influence the propensity to generate WOM, which indirectly promotes blood donation. Thus, it is crucial to invest resources in improving the service quality of blood centres.

Finally, at the micro-level, our results allowed us to understand better individuals’ behaviour related to blood donation for donors and non-donors. Our findings indicate the role of inhibitors that seem to be the strong barrier for non-donors, even though communication and information could support overcoming them in the long term.

### Limitations and future perspectives

Despite the importance of this study’s main findings, some limitations exist and should be overcome by future studies. *First*, the present research was carried out before the COVID-19 pandemic. It could be interesting to repeat the analysis to investigate how and why the pandemic has affected the donation phenomenon, influencing the propensity and promotion of blood donation.

*Second*, both for the donors and non-donors the sample size is consistent with the proposed study’s explorative nature, even if in future research, the sample will be enlarged to enrich the proposed findings. Indeed, the sample only includes Italian citizens. Still, it can be enlarged to other countries since it can help investigate different cultural viewpoints, evidencing the normative differences among Countries. Indeed, as Suemnig et al. [6] stated, the factors that affect behaviours among donors and non-donors can vary based on sociodemographic features such as cultural background (age, gender, etc.). Hence, future research may investigate and compare the phenomenon in different cultural contexts to generalise the factors that encourage citizens to donate over time.

### Supplementary Information

**Additional file 1.**

## Appendix

**Table 9:** In-depth interview topic guide (qualitative survey)

**Donors**	
▪ Tell me about the blood donation phenomenon.	
▪ Strengths and weaknesses of the blood donation phenomenon.	
▪ What are the characteristics that a blood donor should possess?	
▪ Motivations, experiences and satisfaction with donations.	
▪ Tell me about your latest experience as a blood donor.	
▪ Service quality and blood donation. What are the key quality aspects for donors?	
▪ Are you informed about blood donation initiatives? What means of communication should be used to promote donation?	
**Non donors**	
▪ Tell me about the donation phenomenon.	
▪ Strengths and weaknesses of the blood donation phenomenon.	
▪ What are the characteristics that a blood donor should possess?	
▪ The obstacles, pitfalls and shortcomings with donations.	
▪ Service quality and blood donation. What should be the key quality aspects for donors?	
▪ Are you informed about blood donation initiatives? What means of communication should be used to promote donation?	

**Table 10:** Demographic characteristics of the qualitative in-depth interview respondents

	Donors			Non-donors		
ID	***Sex***	***Age***	***Donor***	ID	***Sex***	***Age***
1	F	18–25	Occasional	1	F	18–25
2	F	36–45	Regular	2	M	26–35
3	F	26–35	Occasional	3	F	26–35
4	F	36–45	Occasional	4	M	18–25
5	M	36–45	Regular	5	F	26–35
6	M	36–45	Regular	6	F	26–35
7	M	26–35	Regular	7	F	26–35
8	F	26–35	Occasional	8	M	36–45
9	F	26–35	Occasional	9	M	18–25
10	M	26–36	Occasional	10	M	26–35
11	F	46–55	Regular	11	F	18–25
12	M	36–45	Regular	12	F	18–25
13	M	26–35	Regular	13	F	26–35
14	F	18–25	Regular	14	M	18–25
15	M	36–45	Regular	15	F	36–45

**Table 11:** Distribution of donors’ respondents in Italy Regions

Region	Frequency	Percentage
Veneto	44	25.4%
Piedmont	31	17.9%
Lazio	31	17.9%
Puglia	20	11.6%
Sicily	11	6.4%
Basilicata	10	5.9%
Friuli Venezia Giulia	6	3.5%
Liguria	5	2.8%
Emilia Romagna	3	1.7%
Abruzzo	3	1.7%
Campania	2	1.2%
Calabria	2	1.2%
Valle d’Aosta	1	0.6%
Sardinia	1	0.6%
Reggio Calabria	1	0.6%
Marche	1	0.6%
Lombardy	1	0.6%

**Table 12:** Residual standardized Variances, Donors (Group A) and Non-donors (Group B)

Residual standardized Variances Group A: Donors					Residual standardized Variances Group B: Non-Donors				
Code	Estimate	S.E.	Est./S.E.	P-value	Code	Estimate	S.E.	Est./S.E.	P-value
ATT1	0.527	0.050	10.586	0.000	ATT1	0.527	0.050	10.586	0.000
ATT2	0.467	0.047	10.003	0.000	ATT2	0.467	0.047	10.003	0.000
ATT3	0.449	0.049	9.169	0.000	ATT3	0.449	0.049	9.169	0.000
ATT4	0.416	0.058	7.151	0.000	ATT4	0.416	0.058	7 .151	0.000
ATT5	0.277	0.040	6.850	0.000	ATT5	0.277	0.040	6.850	0.000
ATT6	0.468	0.046	10.096	0.000	ATT6	0.468	0.046	10.096	0.000
SN1	0.526	0.054	9.691	0.000	SN1	0.526	0.054	9.691	0.000
SN2	0.354	0.066	5.344	0.000	SN2	0.354	0.066	5.344	0.000
SN3	0.479	0.057	8.432	0.000	SN3	0.479	0.057	8.432	0.000
SQ2	0.348	0.046	7.575	0.000	SQ2	0.348	0.046	7.575	0.000
SQ3	0.261	0.036	7.218	0.000	SQ3	0.261	0.036	7.218	0.000
SQ5	0.188	0.042	4.513	0.000	SQ5	0.188	0.042	4.513	0.000
PBC1	0.317	0.046	6.898	0.000	PBC1	0.317	0.046	6.898	0.000
PBC2	0.359	0.057	6.309	0.000	PBC2	0.359	0.057	6.309	0.000
PBC3	0.575	0.045	12.899	0.000	PBC3	0.575	0.045	12.899	0.000
INT1	0.182	0.050	3.620	0.000	INT1	0.182	0.050	3.620	0.000
INT2	0.220	0.032	6.769	0.000	INT2	0.220	0.032	6.769	0.000
INT3	0.724	0.043	16.891	0.000	INT3	0.724	0.043	16.891	0.000
VOM4	0.217	0.056	3.856	0.000	VOM4	0.217	0.056	3.856	0.000
VOM5	0.322	0.047	6.916	0.000	VOM5	0.322	0.047	6.916	0.000
COM1	0.189	0.040	4.704	0.000	COM1	0.189	0.040	4.704	0.000
COM2	0.150	0.036	4.130	0.000	COM2	0.150	0.036	4.130	0.000
COM3	0.458	0.060	7.602	0.000	COM3	0.458	0.060	7.602	0.000
COM4	0.518	0.056	9.234	0.000	COM4	0.518	0.056	9.234	0.000
INHI2	0.339	0.056	6.090	0.000	INHI2	0.339	0.056	6.090	0.000
INHI3	0.194	0.042	4.619	0.000	INHI3	0.194	0.042	4.619	0.000
INHI4	0.465	0.050	9.245	0.000	INHI4	0.465	0.050	9.245	0.000
INHI5	0.705	0.049	14.373	0.000	INHI5	0.705	0.049	14.373	0.000
INHI6	0.424	0.059	7.225	0.000	INHI6	0.424	0.059	7.225	0.000
ATT	0.653	0.136	4.813	0.000	ATT	0.653	0.136	4.813	0.000
INHI	0.966	0.023	42.434	0.000	INHI	0.966	0.023	42.434	0.000
INT	0.503	0.065	7.690	0.000	INT	0.503	0.065	7.690	0.000
WOM	0.428	0.070	6.115	0.000	WOM	0.428	0.070	6.115	0.000

## Abbreviations

SEM: Structural Equation Modelling
SDT: Self-determination Theory
TPB: Theory of Planned Behaviour
WOM: Word Of Mouth
e-WOM: electronic WOM
WHO: Word Health Organization
