# Supplementary material for: Promoting the propensity for blood donation through the understanding of its determinants
Source: BMC Health Serv Res. 2021 Feb 8;21:127. doi: 10.1186/s12913-021-06134-8 (PMC7868170; doi:10.1186/s12913-021-06134-8)
Supplement: Supplementary file 1 — Additional file 1. [file 12913_2021_6134_MOESM1_ESM.docx]

| **Dimension** | **Item** | **Code** | **References** |
| --- | --- | --- | --- |
| *Attitude* | I think that donating blood is ethical. | ATT_1 | [11] |
|  | I think that donating blood is useful. | ATT_2 |  |
|  | I think that donating blood is safe. | ATT_3 |  |
|  | I think that donating blood is a moral obligation. | ATT_4 |  |
|  | Donating blood is important to me. | ATT_5 |  |
|  | I think that donating blood is a personal responsibility. | ATT_6 |  |
| *Subjective norm* | Most people that are important to me appreciate that I am a donor. | SN_1 | [11] |
|  | Most people that are important to me think that I should donate blood. | SN_2 |  |
|  | Most people that are important to me appreciate the I donate. | SN_3 |  |
| *Perceived behavioural control* | It is easy to possess the requisites to donate. | PBC_1 | [11] |
|  | People who have a regular life are more likely to be blood donors. | PBC_2 |  |
|  | If I decide to donate in the next weeks, I could do it without difficulty. | PBC_3 |  |
| *Inhibitors* | Fear of needles. | INHI_2 | [16]  Qualitative phase |
|  | Pain when drawing. | INHI_3 |  |
|  | Sight of blood. | INHI_4 |  |
|  | Unpleasant sensations related to the withdrawal (fainting. weakness. nausea). | INHI_5 |  |
|  | Withdrawal preparation (compliance with requirements). | INHI_6 |  |
| *Information and communication* | It is necessary to increase donation awareness activities. | COM_1 | Qualitative phase |
|  | It is necessary to make young people aware of donation through activities in schools and universities. | COM_2 |  |
|  | It is necessary to increase donation awareness through social media and social networking. | COM_3 |  |
|  | It is necessary to introduce promotional campaigns through web and social networks | COM_4 |  |
| *Service quality* | The medical staff should be kind. | SQ_2 | [32,66,67] |
|  | The medical staff should be competent. | SQ_3 |  |
|  | The medical staff should support me during and after the donation. | SQ_5 |  |
| *Intention* | I would like to donate blood again (or in the future). | INT_1 | [11,16] |
|  | I would like to donate blood more often (or for the first time). | INT_2 |  |
|  | I would like to donate blood even without receiving benefits (e.g., discounts, economic benefits). | INT_3 |  |
| WOM | I would recommend blood donation to my friends and family. | WOM_1 | [11,16] |
|  | I would recommend blood donation on social networks. | WOM_2 |  |

**ANNEX I. Questionnaire structure**
